# Can atrial fibrillation ablation outcomes be properly predicted with electrocardiography and artificial intelligence?

**DOI:** 10.1093/ehjdh/ztag029

**Published:** 2026-02-11

**Authors:** Jasper R Vermeer, Richard A J Post, Thomas Mast, Edwin R van den Heuvel, Lukas R C Dekker

**Affiliations:** Department of Cardiology, Catharina Hospital Eindhoven, Michelangelolaan 2, Eindhoven, The Netherlands; Department of Electrical Engineering, Eindhoven University of Technology, Eindhoven, The Netherlands; Department of Biostatistics, Erasmus University Medical Center, Rotterdam, The Netherlands; Department of Epidemiology, Erasmus University Medical Center, Rotterdam, The Netherlands; Department of Cardiology, Catharina Hospital Eindhoven, Michelangelolaan 2, Eindhoven, The Netherlands; Department of Mathematics and Computer Science, Eindhoven University of Technology, Eindhoven, The Netherlands; Department of Cardiology, Catharina Hospital Eindhoven, Michelangelolaan 2, Eindhoven, The Netherlands; Department of Electrical Engineering, Eindhoven University of Technology, Eindhoven, The Netherlands

**Keywords:** Ablation, Atrial fibrillation, Deep learning, Electrocardiogram, Neural Networks, Prediction

## Abstract

**Aims:**

The success of ablation for atrial fibrillation (AF) varies, often leading to repeat ablation. Reliable prediction of repeat ablation remains challenging. This study aimed to investigate if AF ablation outcomes can be predicted with an electrocardiogram (ECG)-based deep learning (DL) algorithm.

**Methods and results:**

We included 865 patients undergoing AF ablation, of whom 163 (18.8%) required a repeat procedure during a minimum follow-up of 572 days. A deep neural network was trained on the raw data of the standard 12-lead ECG obtained within 3 months prior to the index ablation, using stratified nine-fold nested cross-validation. Unfortunately, the model achieved a nested cross-validation area under the receiver operating characteristic curve (AUC) of only 0.61 (95% CI: 0.57–0.64). For comparison, the same analytic approach achieved significantly higher accuracy for sex classification (AUC = 0.87, 95% CI: 0.86–0.89). A random forest model only using clinical variables (age, sex, body mass index, AF pattern) yielded a similar performance for a repeat ablation (cross-validated AUC = 0.59, 95% CI: 0.55–0.63), suggesting limited added value of ECG-based prediction. SHapley Additive exPlanations was used to pinpoint the most relevant ECG segments and highlighted contributions from P-wave, QRS-complex, and T-wave features.

**Conclusion:**

The DL model demonstrated limited ability to predict repeat AF ablation based on the standard 10-second 12-lead ECG. Ablation outcomes may be influenced more by non-ECG parameters or require larger datasets or long-term ECG monitor data, and multi-modality inputs to be accurately predicted.

## Graphical Abstract

**Graphical Abstract:**
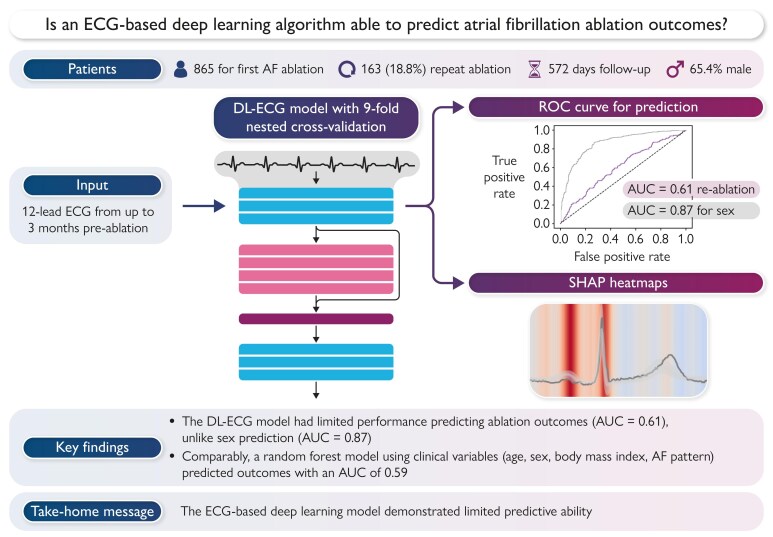

## Introduction

Atrial fibrillation (AF) is the most prevalent heart rhythm disorder and is associated with impaired quality of life, stroke and increased mortality.^1^ The current increasing AF prevalence is accompanied by substantial healthcare costs. One of the pillars in the management of AF is the maintenance of normal sinus rhythm.^2^ This can be achieved with an invasive procedure, called pulmonary vein isolation (PVI). However, up to 20% of patients experience a return of AF within one year after PVI ablation, necessitating the need for a repeat ablation.^3–5^

Identifying patients that are likely to need a repeat ablation potentially benefits shared decision-making as well as allocation of healthcare resources. Follow-up and treatment decisions can be carried out more efficiently in patients if their risk of AF recurrence is more clear. Therefore, various risk models have previously been proposed to predict post-ablation AF-recurrence, involving patient characteristics, and electrocardiogram (ECG) parameters.^6–8^ Unfortunately, these models fail to predict accurate ablation outcomes and are not widely used in clinical practice.

Over the last decade, deep learning (DL) algorithms gained increasing attention in automatic ECG interpretation and clinical prediction models.^9^ Deep neural network (DNN) algorithms have been developed to predict various outcomes, including a patient's sex, the risk of developing AF, and the risk of developing malignant ventricular arrhythmias.^10–14^ This suggests that the standard 12-lead ECG contains complex features that are not immediately apparent to cardiologists.

The application of DL-enabled ECG algorithms for predicting the outcomes of AF ablation is as yet still limited. Previous studies reported reasonable performance, with areas under the receiver operating characteristic (ROC) curves (AUCs) of 0.767 in 156 patients and 0.84 in 1618 patients.^15,16^ However, these algorithms were either trained on augmented data or lacked validation on independent test sets. As a result, it remains uncertain whether ECGs truly contain predictive information on ablation outcomes. Moreover, current DL approaches are often regarded as ‘closed box’ models, unable to point out the relevant features in the ECGs, limiting clinical interpretability.

The aim of this study is to investigate whether pre-ablation ECGs contain sufficient information to predict AF ablation outcomes, and if so, to develop an ECG-based DL prediction model that can support clinicians in shared decision-making for initial ablation candidates and their follow-up. Furthermore, this study seeks to provide interpretable insights into how AF recurrence risk is reflected in the ECG.

## Methods

### Study design

This is a retrospective cohort study carried out in the Catharina Hospital in Eindhoven, the Netherlands, a tertiary referral hospital for ablation of AF. All patients who underwent their first PVI ablation between 2016 and 2020 and who were 18 years or older at the time of ablation were included. Patients were excluded if they previously underwent non-AF ablation or had complete bundle branch block before the index AF ablation. Furthermore, patients were excluded if they did not have their follow-up planned in the Catharina Hospital or if no ECG of reasonable quality was available within 3 months before ablation. Patients were excluded if no ECG in sinus rhythm and without pacing was available. The responsible medical ethical committee (MEC-U) issued a waiver for informed consent (W20.271).

### Ablation procedure and outcomes

Various ablation methods were used to achieve PVI, including cryoballoon and point-by-point radiofrequency ablation. Patients were followed up in the outpatient clinic at 3 months and 1 year after ablation. At this point, rhythm was assessed with a standard 12-lead ECG. If patients experienced AF complaints, a Holter monitor was used to confirm the presence of AF. Patient could undergo a repeat ablation if they had confirmed AF with symptoms more than three months after the index ablation. However, patient could decide to not undergo a repeat ablation if they experienced enough improvement in AF symptoms and improvement of quality of life. We focused on the outcome repeat ablation, because this patient-relevant endpoint is unequivocally representing clinical relevance.^17^

### Data extraction

Baseline characteristics and ablation outcomes were retrieved from medical records using the standards of the Netherlands Heart Registry (NHR). The NHR is a nationwide quality registry, which has a certified data quality control system to ensure completeness and quality of data.^18^

We based our analysis on the most recent ECG that was available in the three months before ablation. ECG data were recorded at 250 Hz, containing 10 s of the 12 standard leads. We excluded leads III, aVL, aVR, and aVF from the model, as they are mathematically derived from leads I and II and would not contribute any additional information. The MUSE Marquette™ 12SL™ ECG Analysis Program was used to determine median ECGs. First, the program aligned all QRS complexes in the 10-second ECG of the same shape (e.g. excluding premature ventricular complexes or pacemaker complexes). Alignment was done for all leads simultaneously using QRS detection points. Then, for each data point the median voltage was determined. Next, the algorithm generates a representative QRS complex from the median voltages that are found at each successive sample time. The final median-beat vector was 300 (sampled at 250 Hz * 1.2 s). Specific amplitude normalization was not performed. However, high- and low- band filters were used to correct for baseline drift before determining the location of the QRS complexes. The median recordings, consisting of the averaged waveforms of the QRS complexes in the ECG, were used as input for the network.

### Algorithm development and evaluation

We employed an architecture known as a residual DNN, which has been successfully used for arrhythmia detection.^9,19^ Rather than comparing multiple network architectures, we deliberately evaluated whether this previously published architecture could be transferred to the task of predicting repeat ablation. The network architecture is presented in Supplementary material online, *Figure S1* in the Supplementary material; in this work, only one residual layer was used. Fixed hyperparameters included a kernel size of 16, batch size of 16, and a dropout rate of 0.6. The networks were trained using the Adam optimizer with a learning rate of 0.01 and L2 regularization penalty of 0.1, minimizing the binary cross-entropy loss on the training data while using validation loss for early stopping and model selection. Each DNN was trained until the validation loss did not improve for 12 epochs, and the fit with the lowest validation loss was returned. Model development followed stratified (on outcome) nine-fold nested cross-validation. For each outer fold, eight models with identical architecture and hyperparameters were trained on the remaining data, each using a different inner fold for validation and early stopping. Predictions from these eight models were averaged to evaluate the corresponding outer-test fold, resulting in a total of 72 trained models. Because the fitted model and optimal early-stopping point depended strongly on the particular validation fold, we averaged predictions across the inner-fold models rather than refitting a single model on the full outer-training set. To reduce stochastic variability in training, each inner-fold model was trained 20 times with different random initializations, and the run with the lowest validation loss was retained; these repetitions did not increase the number of models included in the ensemble. The fixed hyperparameters were selected from a predefined grid by repeating the full nested cross-validation procedure for each hyperparameter combination, but only with one random initialization. The grid consisted of kernel size {8, 16}, batch size {16, 32}, dropout rate {0.2, 0.6}, learning rate {0.001, 0.01}, and L2 regularization penalty {0, 0.1}. The final configuration was chosen based on the lowest average binary cross-entropy on the validation folds as presented in Supplementary table S1. A sensitivity analysis was performed to verify that increasing the number of residual layers did not improve the predictive performance.

Predictive performance was summarized using ROC curves derived from three sets of predictions. The test ROC was based on 865 predictions, one for each individual in the outer test fold, obtained from the ensemble of eight DNNs trained on the remaining folds. The validation ROC was based on predictions for individuals when their data was part of the validation fold of the inner cross-validation during model development. Because each individual’s data did belong once to the validation fold within each outer cross-validation cycle, this resulted in eight validation predictions per individual, yielding a total of 865 × 8 = 6920 validation predictions. Finally, the training ROC was based on predictions for individuals when their data were used for model training, that is, the predictions obtained from the 8 × 7 = 56 models in which each individual was included in the training dataset, resulting in 865 × 56 = 48 440 training predictions in total. To assess the robustness of our DNN approach and the quality of the ECG data, we developed a similar prediction model for the outcome sex. This straightforward endpoint allows us to verify whether the methods and data are of sufficient quality to support reliable predictive modelling. We used SHapley Additive exPlanations (SHAP) to compute time-resolved importance values for each ECG. SHAP estimates how much each input feature contributes to the model’s output by comparing the prediction for the observed sample with predictions obtained when that feature is replaced by values drawn from a reference distribution.^20^ In our implementation, SHAP values were computed for each time point of each ECG lead, yielding a localized importance map highlighting waveform regions that influenced the model’s prediction. Importantly, SHAP values were calculated using predictions from the ensemble of models, in which the individual ECG was not used for training. ECGs from patients without repeat ablation served as the reference population. Finally, to study the gain in performance moving from ECG summary statistics to full ECG data, we also fitted a random forest using the ECG parameter features available from MUSE (ventricular rate, atrial rate, PR interval, QRS duration, QT interval, QT corrected, P axis, R axis and T axis), using nine-fold cross-validation. Although not the focus of this study, we have additionally considered clinical features (paroxysmal or persistent AF, sex, age, and body mass index) in the random forest models. Random forest models were fitted for all subsets of considered features and we presented the subset leading to the highest nine-fold cross-validated AUC. Because the goal was to evaluate whether a modern DL architecture could provide additional insight beyond a standard baseline, and not to optimize classical machine-learning models, only a random forest model was used as a representative comparator. The DNN analyses were performed using Python and the training scripts can be found at https://github.com/RAJP93/CZH_reablation.

### Statistical analysis

Diagnostic performance measures included the AUC, positive predictive value (PPV), negative predicted value (NPV) sensitivity, specificity, F1 score and accuracy. Descriptive statistics are used to describe baseline characteristics. Continuous variables are presented with a mean ± standard deviation or median with interquartile range depending on the distribution. Differences in categorical variables between the PVI outcome groups are tested using the Chi-square test or Fisher’s exact test (as appropriate). For continuous variables the Student *t* or the Mann–Whitney U is used to compare outcome groups for non-normally distributed variables.

## Results

### Overall procedure summary

**Figure 1:**
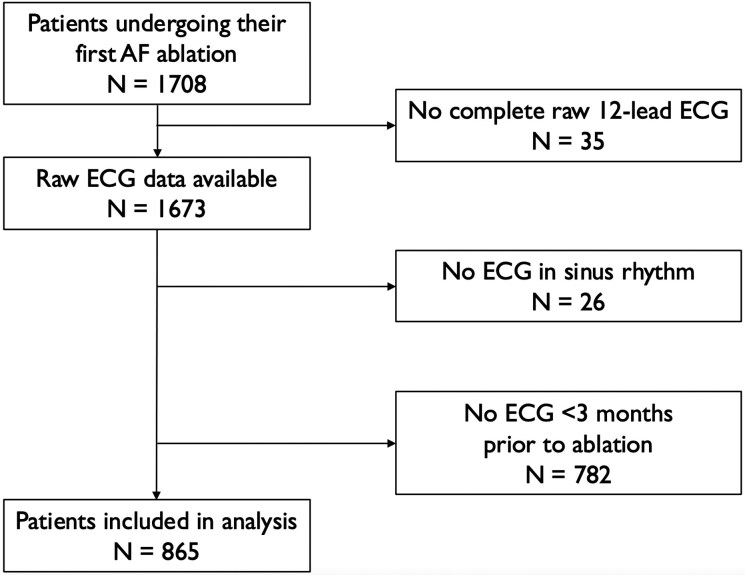
Patient inclusion flowchart.

The patient inclusion flowchart is displayed in *Figure 1*. In our cohort, 1,708 patients underwent their first PVI for AF. However, 35 patients did not have any full 12-lead raw data ECG available in their patient files and were not included. Of the remaining 1,673 patients, 808 were excluded from our analysis: 26 did not have an ECG in sinus rhythm and 782 did not have an ECG within 3 months prior to ablation. Finally, a total of 865 patients were included in the present analysis. The median age of study participants was 64.1 [56.7;70.1] years; 566 (65.5%) were male. A total of 163 (18.8%) patients received a repeat ablation. Minimum follow-up was 572 days. Baseline characteristics are displayed in *Table 1*.

**Table 1:** Baseline characteristics

	Repeat ablation	No repeat ablation	*P*-value
	*N* = 163	*N* = 702	
Sex (male)	95 (58.3%)	471 (67.2%)	**0**.**039^a^**
Age (years)	64.9 [56.3;71.1]	64.1 [56.7;69.9]	0.380^b^
Age class			0.605**^a^**
−<60 years	57 (35.0%)	253 (36.0%)	
−60–69 years	60 (36.8%)	277 (39.5%)	
−≥70 years	46 (28.2%)	172 (24.5%)	
BMI (kg/m^2^)	27.2 [24.8;30.4]	26.8 [24.3;29.1]	0.058^b^
BMI weight class			**0.037^a^**
−normal (<25)	41 (25.2%)	198 (29.0%)	
−overweight (25–29.9)	74 (45.4%)	347 (50.8%)	
−obese (≥30)	48 (29.4%)	138 (20.2%)	
AF type			**0.003** ^c^
−paroxysmal	107 (67.3%)	557 (79.9%)	
−persistent	49 (30.8%)	132 (18.9%)	
−longstanding-persistent	3 (1.89%)	8 (1.15%)	
LVEF			**0.048** ^c^
−good (≥50%)	128 (83.7%)	572 (89.8%)	
−moderate (30–49%)	23 (15.0%)	62 (9.73%)	
−severe (<30%)	2 (1.31%)	3 (0.47%)	
Creatinine (µmol/L)	83.0 [74.2;93.8]	85.0 [74.0;97.0]	0.701^b^
CHA2DS2-VASc			**0.024** ^c^
0	31 (19.6%)	149 (22.4%)	
1	39 (24.7%)	180 (27.1%)	
2	32 (20.3%)	181 (27.2%)	
3	36 (22.8%)	89 (13.4%)	
4	14 (8.86%)	47 (7.07%)	
5	5 (3.16%)	14 (2.11%)	
6	0 (0.00%)	5 (0.75%)	
7	1 (0.63%)	0 (0.00%)	

AF, atrial fibrillation; BMI, body mass index; LVEF, left ventriculair ejection fraction.

^a^Pearson's Chi-squared test.

^b^Wilcoxon rank sum test.

^c^Fisher's exact test.

The baseline characteristics of the excluded patients did not differ significantly from those of the included patients, except that the proportion of persistent AF was higher among the excluded patients (*P* < 0.001), and median age was higher in excluded patients (64.8 vs. 64.1 years; *P* = 0.027). Baseline characteristics of excluded patients are displayed in Supplementary material online, *Table S2*.

### Prediction from clinical features and ECG parameter features

The random forest fits achieved a nine-fold cross-validation AUC of 0.57 (95% CI: 0.51–0.63) using only ECG features, 0.59 (95% CI: 0.55–0.63) using only clinical features, and 0.60 (95% CI: 0.55–0.65) using both, as presented in *Table 2*.

**Table 2:** Random forest model nine-fold cross-validation fit performance summary for repeat-ablation and sex prediction. The PPV, NPV, Sensitivity, Specificity, F1, and Accuracy are based on threshold probabilities of 0.25 (*), 0.275 (**), or 0.5 (***)

	AUC	PPV	NPV	Sensitivity	Specificity	F1	Accuracy
*Repeat ablation:*							
Clinical features*	0.59	0.25	0.83	0.28	0.81	0.26	0.71
ECG parameter features**	0.57	0.22	0.82	0.31	0.75	0.25	0.66
Clinical features + ECG parameter features**	0.60	0.24	0.82	0.28	0.78	0.26	0.69
*Sex:*							
ECG parameter features***	0.73	0.75	0.63	0.86	0.45	0.80	0.72

### DL-enabled ECG algorithm outcomes

**Figure 2:**
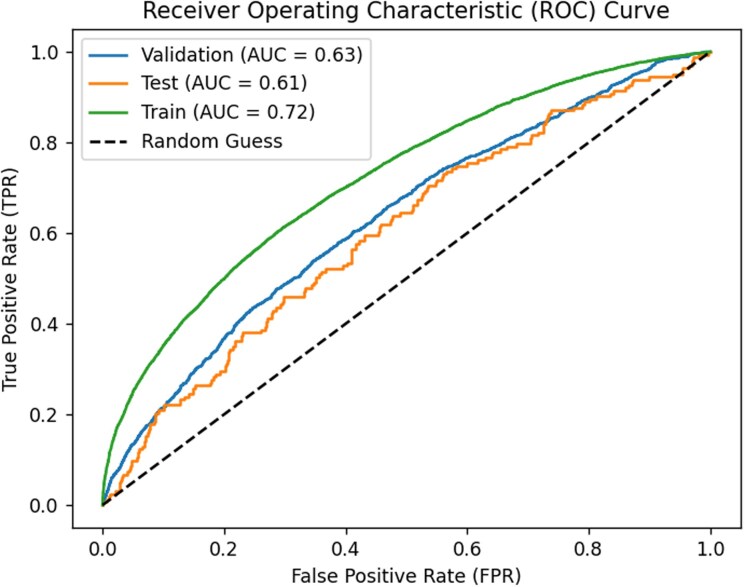
Receiver-operator curve for the predicted probability of repeat ablation within 572 days. Curves are based on the predicted probability for a patient based on the residual DNN models where the patient’s data was not used for fitting (test, 865 predictions), was used in the validation dataset (865 × 8 × 1), or was used in the training dataset (865 × 8 × 7), respectively.

Using raw electrogram signals only, our DNN achieved a nested nine-fold cross-validation AUC of 0.61 (95% CI: 0.57–0.64) for predicting repeat ablation based on the last median ECG within 3 months before the ablation surgery, as presented in *Table 3*. When predicting sex, the DNN achieved an AUC of 0.88 (see Supplementary material online, *Table S3* and Supplementary material online, *Figure S3*, 95% CI: 0.86–0.89). The performance statistics for predictions based on models for which a patient was used in the validation data or training data can be found in Supplementary material online, *Tables S5* and *S6*, respectively. The receiver-operator curve for repeat ablation prediction based on the models for which a patient was used in the test, validation, or training data, respectively, is presented in *Figure 2*. The precision-recall curve is presented in Supplementary material online, *Figure S2*. The ensemble of trained DNNs is publicly available via https://github.com/RAJP93/CZH_reablation. Based on the model trained on the other eight-folds, 14% of the patients had a predicted probability of repeat ablation higher than 0.275, and the prevalence of repeat ablation within 572 days in this subgroup was 30% and significantly higher than in the whole cohort (*P* = 0.002).

**Table 3:** Neural net (nested) cross-validation performances summary. The PPV, NPV, Sensitivity, Specificity, F1 and Accuracy are presented for a repeat ablation threshold probability of 0.275

Fold	AUC	PPV	NPV	Sensitivity	Specificity	F1	Accuracy
*1*	0.52	0.33	0.82	0.16	0.92	0.21	0.77
*2*	0.55	0.31	0.83	0.22	0.89	0.26	0.76
*3*	0.62	0.23	0.82	0.17	0.87	0.19	0.74
*4*	0.71	0.41	0.86	0.39	0.87	0.40	0.78
*5*	0.54	0.30	0.83	0.17	0.91	0.21	0.77
*6*	0.63	0.43	0.83	0.17	0.95	0.24	0.80
*7*	0.64	0.27	0.84	0.39	0.76	0.32	0.69
*8*	0.61	0.22	0.82	0.22	0.82	0.22	0.71
*9*	0.61	0.29	0.82	0.11	0.94	0.16	0.78
**Overall**	**0**.**61**	**0**.**30**	**0**.**83**	**0**.**22**	**0**.**88**	**0**.**25**	**0**.**76**

We have also repeated the analysis based on the first median ECG within 1 month after ablation. The nested nine-fold cross-validation AUC is similar and equal to 0.62 (95% CI: 0.59–0.67) based on a cohort of *n* = 1222 patients (see Supplementary material online, *Table S4* and Supplementary material online, *Figure S4*).

### Heatmaps of the ECG

**Figure 3:**
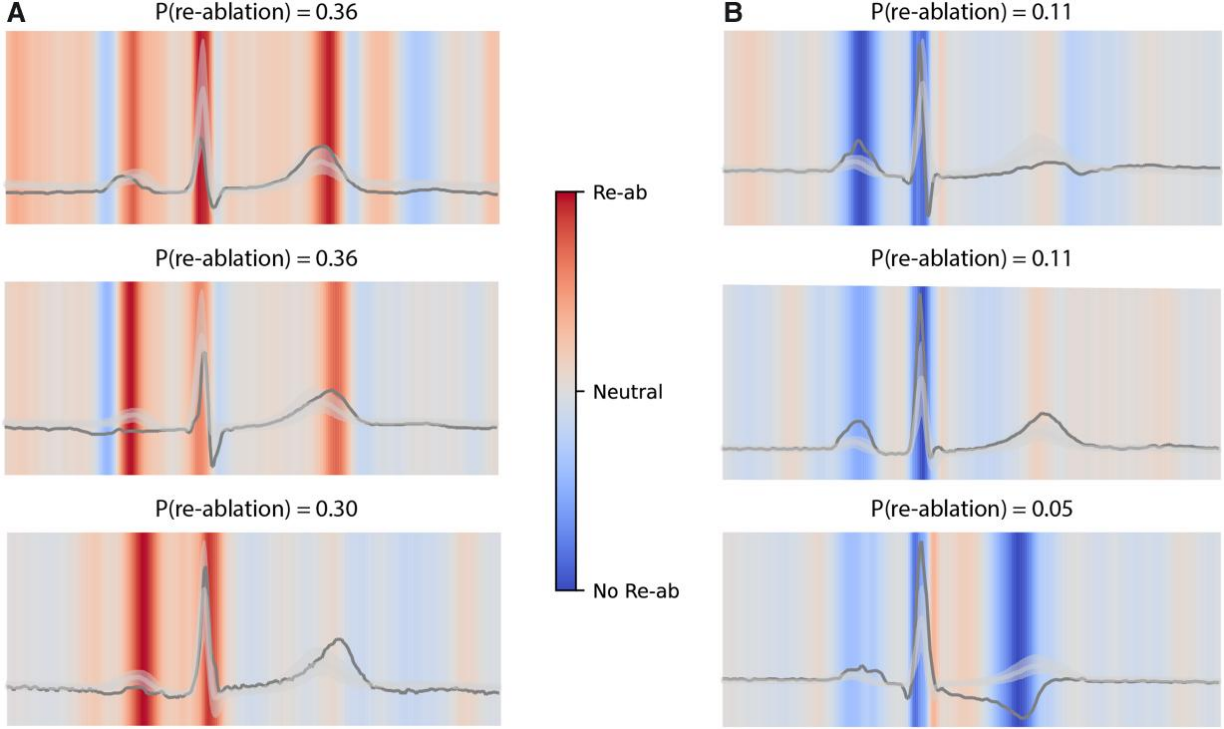
ECG heatmaps. SHAP heatmaps were used to identify which segments in the ECG most influenced the prediction of the model per patient. The reference to contextualize importance scores was ECGs from patients without repeat ablation. The median, 25%, and 75% pointwise quantiles of the ECGs from patients without repeat ablation are represented by the grey line and transparent band. The scores were averaged across leads and models to generate a robust temporal importance heatmap. Examples of higher (*A*) and lower (*B*) risk for repeat ablation patients are presented with their lead-I median ECGs shown as black lines. In the upper left panel, both QRS and T-wave were important for predicting repeat ablation in that specific patient. In the upper right panel, both P-wave and QRS were important for predicting no repeat ablation in a different patient.

SHAP heatmaps provided a qualitative visualization of the waveform regions influencing the model’s predictions. Dark red areas revealed regions associated with an increased predicted probability of repeat ablation, while dark blue areas indicated regions associated with a decreased probability. *Figure 3* displays examples of these heatmaps. When reviewing heatmaps across all individuals, contributions were observed across multiple parts of the ECG cycle, although no consistently dominant pattern emerged. At a general level, activity within the P-wave, QRS complex, and T-wave regions appeared to contribute to the model’s predictions. We did not observe clear evidence for strong interaction effects between these components in the SHAP visualizations. Given the model’s overall limited predictive performance, these observations should be considered exploratory.

## Discussion

In this study, we investigated the usefulness of a DL algorithm to predict the risk of repeat ablation within 572 days after a first PVI for AF. We designed and trained an ensemble of DNNs with moderate prediction performance (nine-fold nested cross-validation AUC = 0.61). This was a small improvement compared to prediction using a random forest based on ECG parameters characteristics (AUC = 0.57), or clinical features (AUC = 0.59), or the combination (AUC = 0.60), suggesting that ECG-ML techniques have a limited role in the prediction of repeat ablation, compared to the use of clinical features. Furthermore, in contrast to the sex prediction algorithm, there was much more variability in test AUCs, ranging from 0.52 to 0.71 over the different folds (despite being stratified on outcome), which hints towards heterogeneity in the association of median ECGs and repeat ablation. Precision–recall analysis yielded the same conclusions and demonstrated that predicted-positive individuals had only modestly higher outcome prevalence than the overall population, except at very low recall levels (see Supplementary material online, *Figure S2*). The absence of a steep increase in the ROC curves at low false-positive rates suggests that the model does not identify a subgroup of patients for whom predictions are highly accurate. Consistently, the precision–recall curves show no plateau or sustained region of high precision at non-negligible recall, indicating that the model does not reliably identify a subgroup of reasonable size with high predictive accuracy. The sensitivity analysis with a predictive model using the first median ECG within a month after ablation had similar results, illustrating that the post-ablation ECG did not contain much more information than the pre-ablation ECG.

In contrast, when predicting sex using ECG signals, the same architecture achieved excellent outcomes, illustrating that the training of our neural networks was implemented correctly and that ECG signals were properly recorded. For sex prediction, the median ECG did contain more information than the ECG parameters alone (random forest-based AUC = 0.73). Sex prediction was included solely to verify that our implementation of the modelling pipeline on our ECG recordings could recover a known signal, and was not intended as evidence that the model would perform well on the more challenging task of predicting repeat ablation. The results highlight that classifying repeat ablation from ECGs is more complex than classifying sex from ECGs. We postulate that the mechanisms of AF leading to repeat ablation are heterogeneous, with varying arrhythmogenic mechanisms, substrates and triggers.^21^ It is possible, that a larger number of training ECGs is required to detect the subtle variations within the signals.

The prediction of ablation outcomes has been a research topic for many years due to persistently high rates of AF recurrence. Early efforts to predict outcomes primarily explored clinical criteria, such as left atrial size.^22^ Over time, various risk score models were developed to predict the return of AF after ablation, incorporating clinical characteristics such as age, sex, kidney function, body mass index, left atrial enlargement, and type of AF.^23–28^ However, these risk models performed poorly and had limited clinical utility.^6^ With emerging statistical and computational possibilities, the availability of more advanced AI algorithms has enabled the integration of more complex clinical characteristics into prediction models. The scope of predictive features has been expanded to include imaging information, such as atrial appendage volume determined by computed tomography and atrial fibrosis determined with late gadolinium enhanced magnetic resonance imaging.^29,30^

Over the past decade, the ECG demonstrated increasing potential to be used in AI-algorithms. Studies have demonstrated the possibility of accurately predicting sex and age from ECG data.^13^ Additionally, earlier ECG-based AI tools have successfully predicted conditions such as AF in patients with sinus rhythm, aortic valve stenosis, and malignant ventricular arrhythmias.^10–12,31^

### Electrocardiograph parameters

In the context of AF ablation, several preselected ECG markers, including P-wave parameters have been proposed as predictors of AF recurrence. Salah *et al*. identified pre-ablation P-wave duration and P-wave dispersion as significant predictors of AF recurrence after ablation.^32^ Furthermore, a 1 month after ablation decrease in combined P-wave duration and amplitude was strongly related to freedom from AF after ablation.^8^ A meta-analysis involving 4175 patients further established P-wave duration as an independent predictor of AF return after ablation, revealing that high-risk patients with a P-wave higher than 150 ms had an odds ratio of 10.9 for recurrence following ablation.^7^ Moreover, Li *et al*. found several P-wave-related indices, including P-wave dispersion, P-wave terminal potential and P-wave duration, that were associated with recurrent AF after ablation.^29^

ECG parameters during AF can also serve as predictors of ablation outcomes. For instance, fine AF has worse outcomes than coarse AF. An F-wave amplitude of less than 0.05 mV in lead V1 has been shown to predict return of AF after ablation.^33,34^ Similarly, a shorter AF cycle length on ECG has been linked to a higher likelihood of the return of AF.^35^ Combining parameters that reflect AF complexity, such as dominant frequency and F-wave amplitude, has led to the development of predictive models with significant accuracy. One such model achieved an AUC of 0.79 by incorporating these factors.^36^ Another model, which combined dominant frequency with spatiotemporal variability values from different precordial leads, reached an AUC of 0.94.^37^ Subsequently, it was confirmed that F-wave amplitude in lead V1 and dominant frequency in lead I are effective predictors of long-term recurrence of atrial arrhythmias in patients with non-paroxysmal AF.^38^ However, the performance of all these models was not assessed using cross-validation, increasing the risk of overfitting and making it impossible to compare performance with our algorithm. The small sample sizes further suggest that patients were highly selected and may not be representative of routine clinical practice. Similarly, in our study, certain patient subsets achieved substantially higher AUCs.

The surge in ML-ECG algorithms in cardiology reflects the limited predictive value of classical models. Using random forest machine learning techniques with cross-validation on clinical and 804 preselected ECG parameters, return of AF in 3 years after ablation was predicted with moderate accuracy (AUC = 0.74).^39^ In a different study, by using ECG data of only 156 patients, a CNN using cross-validation achieved an AUC of 0.77 for predicting AF recurrence within 1 year. When ECG data were combined with clinical features and intracardiac electrograms, the AUC improved to 0.86.^15^ Remarkably, in the latter study CHA2DS2-VASc score did already have an AUC of 0.65, which was a better performance than in our study cohort (AUC of 0.55). Similarly, previous studies using a CNN with ECG data effectively predicted the risk of recurrence in patients with paroxysmal AF after ablation with AUC =0.84 and AUC = 0.796.^16,40^ However, both these studies lacked the use of cross-validation or interpretable heatmaps, which leaves its usability in clinical practice to be questioned. Those trained prediction algorithms are not available for other researchers, making it impossible to validate the performance in other cohorts.

### Network generalizability

Cross-validation of the neural network is an essential step in the development of DL algorithms. In our application, the AUCs in the different test folds ranged from 0.52 to 0.71. This highlights that a procedure in which one first randomly draws a test dataset to validate a model afterwards can be highly misleading when studying samples of our size. It will be much more likely that research that by chance selected the fold with test AUC equal to 0.70 will be published than for the one with test AUC equal to 0.52. Being aware of this treat is even more important as trained machine learning models are rarely made publicly available, making it impossible for other researchers to validate the prediction models. However, it should be noted that the application of cross-validation provides an unbiased internal performance estimate, while external validation (independent cohort, temporal hold-out, or leave-one-site-out across institutions or devices) is required to assess generalization to other populations.

### Limitations

Our study has several limitations. First, this is a retrospective single-centre study. Second, the outcome repeat ablation is a subjective endpoint that depends to a great extent on shared decision-making of the clinician and patient. This may explain why the predictive value of our models that are solely based on ECGs and patient characteristics, omitting information about the patients’ quality of life, would provide only moderate prediction performance. Third, the model was not validated externally, limiting its value for other ablation sites. Last, we were not able to include information on anti-arrhythmic medication use, which could also greatly affect ECGs characteristics and decision to perform a repeat ablation.

### Strengths

The strength in our study relies in the robust methodology used, ensuring that our DL model did not overfit using nested cross-validation. As previously mentioned, many ECG-derived DL algorithms are a ‘closed box’, and do not provide insight into the relevant features of the ECG. However, we developed heatmaps that provided information on what part of the ECG is most attributive for our prediction models. This is the first study to use explainable DNNs trained with ECGs for prediction of repeat ablation. Unfortunately, as the algorithm did not perform well, the results of the heatmaps were also limited. Furthermore, the intention of our algorithm was that it is easily implanted in clinical practice. Only the standard 12-lead ECG, and no ECG recordings over multiple hours, were required.

### Clinical perspective

The availability of new statistical methods and DL techniques allows for further increase in knowledge and application of the ECG, and implementation of novel applications such as risk stratification and prediction. We present the development and validation of a machine learning algorithm that aims to predict ablation success. However, for now, we should consider that there is a limited role for AI in predicting ablation outcomes. AF is a heterogeneous disease with varying substrates and triggers among patients, possibly negatively affecting the predictive value of the ECG. Future research should involve the development of an algorithm based on more patients and ECGs, in order to overcome this heterogeneity.

## Conclusion

Our ECG-based DL model demonstrated limited predictive ability for repeat AF ablation. This finding underscores the complexity of predicting AF ablation outcomes, and suggests that ECGs alone may not contain enough information for high prediction power. AF ablation outcomes appear to be driven by multifactorial and largely non-ECG-based determinants. Future efforts to predict ablation outcomes should include more ECGs or move beyond ECG-only approaches and explore multimodal strategies that integrate clinical, imaging, and electrophysiological data to improve predictive performance.

## Supplementary Material

ztag029_Supplementary_Data

## Data Availability

The data underlying this article will be shared on reasonable request to the corresponding author.
