# Supplementary material for: Can atrial fibrillation ablation outcomes be properly predicted with electrocardiography and artificial intelligence?
Source: Eur Heart J Digit Health. 2026 Feb 11;7(2):ztag029. doi: 10.1093/ehjdh/ztag029 (PMC12930191; doi:10.1093/ehjdh/ztag029)
Supplement: ztag029_Supplementary_Data [file ztag029_supplementary_data.docx]

**Supplementary Material**

**Figure S1: Residual deep neural network architecture**


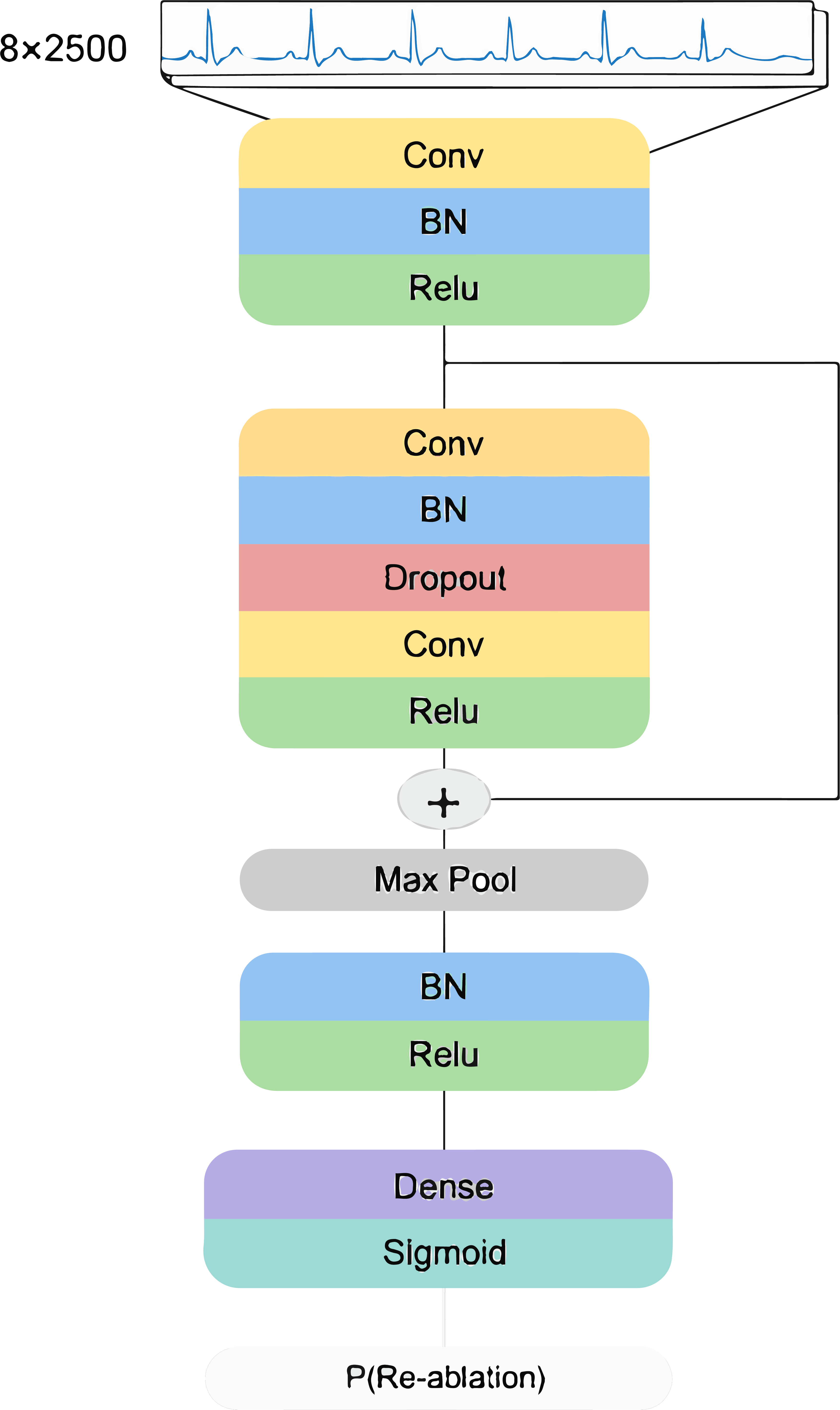


**Figure S2: Precision-recall curve for the predicted probability of repeat ablation within 572 days.** Curves are based on the predicted probability for a patient based on the residual DNN models where the patient’s data was not used for fitting (test, 865 predictions), was used in the validation dataset (865x8x1), or was used in the training dataset (865x8x7), respectively.


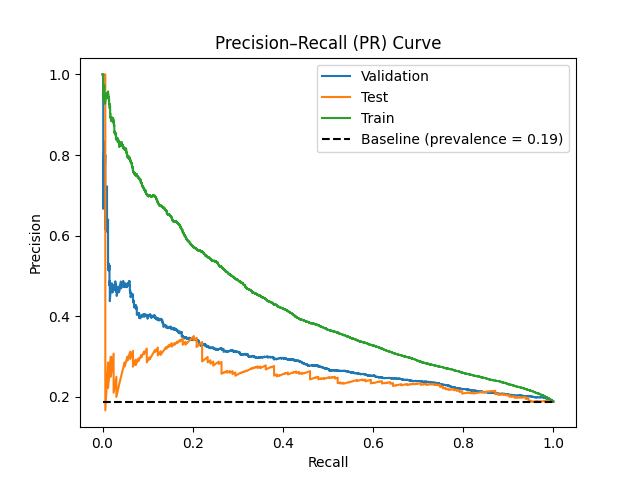


**Figure S3: Receiver-operator curve for sex classification.** Curves are based on the predicted probability for a patient based on the the residual DNN models where the patient’s data was not used for fitting (test, 865 predictions), was used in the validation dataset (865x8x1), or was used in the training dataset (865x8x7), respectively.


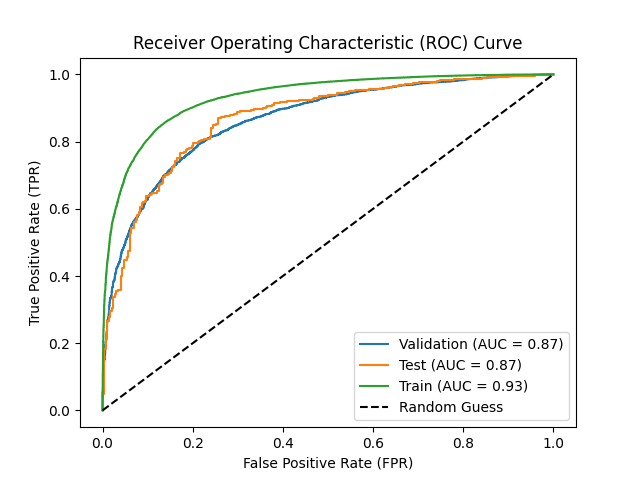


**Figure S4: Receiver-operator curve for the predicted probability of repeat ablation within 572 days given ECG within 1 month after ablation.** Curves are based on the predicted probability for a patient based on the residual DNN models where the patient’s data was not used for fitting (test, 1222 predictions), was used in the validation dataset (1222x8x1), or was used in the training dataset (1222x8x7), respectively.


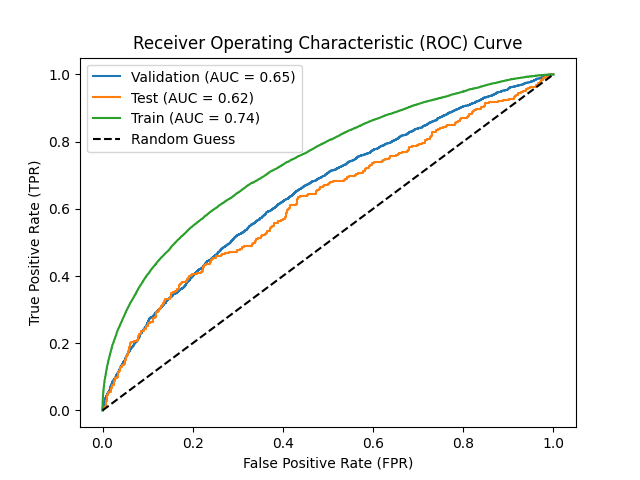


Table S1: Grid search results for hyperparameter turning of the neural network. Configurations vary by kernel size, batch size, dropout rate, learning rate, and L2 regularization penalty. Reported values correspond to the binary cross-entropy loss averaged over the validation folds for the DNN with one residual layer. The final confirmation was selected by minimizing the validation loss.

| **Kernel size** | **Batch size** | **Learning rate** | **Dropout rate** | **L2 penalty** | **Validation loss** | **Training loss** |
| --- | --- | --- | --- | --- | --- | --- |
| 16 | 16 | 0.010 | 0.6 | 0.1 | 0.472 | 0.461 |
| 16 | 16 | 0.010 | 0.2 | 0.1 | 0.476 | 0.465 |
| 8 | 16 | 0.010 | 0.6 | 0.1 | 0.477 | 0.465 |
| 8 | 16 | 0.010 | 0.2 | 0.1 | 0.479 | 0.453 |
| 16 | 16 | 0.010 | 0.6 | 0.0 | 0.480 | 0.467 |
| 16 | 32 | 0.010 | 0.6 | 0.1 | 0.481 | 0.444 |
| 8 | 16 | 0.010 | 0.6 | 0.0 | 0.482 | 0.464 |
| 16 | 32 | 0.010 | 0.2 | 0.1 | 0.482 | 0.443 |
| 8 | 32 | 0.010 | 0.6 | 0.1 | 0.483 | 0.463 |
| 8 | 32 | 0.010 | 0.2 | 0.1 | 0.485 | 0.432 |
| 16 | 16 | 0.010 | 0.2 | 0.0 | 0.487 | 0.461 |
| 16 | 32 | 0.010 | 0.6 | 0.0 | 0.487 | 0.450 |
| 8 | 16 | 0.010 | 0.2 | 0.0 | 0.488 | 0.469 |
| 8 | 32 | 0.010 | 0.6 | 0.0 | 0.489 | 0.483 |
| 16 | 16 | 0.001 | 0.2 | 0.1 | 0.490 | 0.390 |
| 16 | 16 | 0.001 | 0.2 | 0.0 | 0.492 | 0.385 |
| 16 | 32 | 0.010 | 0.2 | 0.0 | 0.492 | 0.448 |
| 16 | 32 | 0.001 | 0.2 | 0.0 | 0.493 | 0.308 |
| 16 | 32 | 0.001 | 0.2 | 0.1 | 0.493 | 0.302 |
| 8 | 16 | 0.001 | 0.2 | 0.0 | 0.495 | 0.370 |
| 8 | 16 | 0.001 | 0.2 | 0.1 | 0.495 | 0.370 |
| 8 | 32 | 0.010 | 0.2 | 0.0 | 0.496 | 0.446 |
| 8 | 32 | 0.001 | 0.2 | 0.1 | 0.498 | 0.295 |
| 8 | 32 | 0.001 | 0.2 | 0.0 | 0.499 | 0.300 |
| 16 | 16 | 0.001 | 0.6 | 0.0 | 0.501 | 0.407 |
| 16 | 16 | 0.001 | 0.6 | 0.1 | 0.505 | 0.404 |
| 16 | 32 | 0.001 | 0.6 | 0.1 | 0.505 | 0.343 |
| 16 | 32 | 0.001 | 0.6 | 0.0 | 0.508 | 0.360 |
| 8 | 32 | 0.001 | 0.6 | 0.0 | 0.512 | 0.358 |
| 8 | 32 | 0.001 | 0.6 | 0.1 | 0.512 | 0.360 |
| 8 | 16 | 0.001 | 0.6 | 0.0 | 0.513 | 0.394 |
| 8 | 16 | 0.001 | 0.6 | 0.1 | 0.513 | 0.388 |

Table S2: Baseline characteristics of excluded patients

|  | Included patients | Excuded patients | *P*-value |
| --- | --- | --- | --- |
|  | N=865 | N=808 |  |
|  |  |  |  |
| Sex (male) | 566 (65.5%) | 563 (69.8%) | 0.071 ^+^ |
| Age (years) | 64.1 [56.7;70.1] | 64.8 [58.0;71.3] | **0.027 ^#^** |
| BMI (kg/m^2^) | 26.9 [24.4;29.4] | 26.7 [24.5;29.6] | 0.754 ^#^ |
| BMI weight class |  |  | 0.770 ^+^ |
| -normal (<25) | 239 (28.3%) | 231 (29.2%) |  |
| -overweight (25-29.9) | 421 (49.8%) | 379 (48.0%) |  |
| -obese (≥30) | 186 (22.0%) | 180 (22.8%) |  |
| AF type |  |  | **<0.001** ^ |
| -paroxysmal | 664 (77.6%) | 487 (61.0%) |  |
| -persistent | 181 (21.1%) | 307 (38.5%) |  |
| -longstanding-persistent | 11 (1.29%) | 4 (0.50%) |  |
| LVEF |  |  | 0.340 ^+^ |
| -good (≥50%) | 700 (88.6%) | 645 (86.7%) |  |
| -moderate (30-49%) | 85 (10.8%) | 90 (12.1%) |  |
| -severe (<30%) | 5 (0.63%) | 9 (1.21%) |  |
| Creatinine (µmol/L) | 84.0 [74.0;96.0] | 85.0 [75.0;97.0] | 0.601 ^#^ |
| CHA2DS2-VASc |  |  | 0.826 ^ |
| 0 | 180 (21.9%) | 170 (21.8%) |  |
| 1 | 219 (26.6%) | 188 (24.1%) |  |
| 2 | 213 (25.9%) | 214 (27.4%) |  |
| 3 | 125 (15.2%) | 112 (14.3%) |  |
| 4 | 61 (7.41%) | 64 (8.19%) |  |
| 5 | 19 (2.31%) | 23 (2.94%) |  |
| 6 | 5 (0.61%) | 6 (0.77%) |  |
| 7 | 1 (0.12%) | 3 (0.38%) |  |
| 8 | 0 (0.00%) | 1 (0.13%) |  |

AF, atrial fibrillation; BMI, body mass index; LVEF, left ventriculair ejection fraction

**^+^** Pearson's Chi-squared test
^#^ Wilcoxon rank sum test
^ Fisher's Exact test

Table S3: Neural Net (Nested) Cross-validation Performances Summary for sex prediction. The PPV, Sensitivity, Specificity, F1, and Accuracy are presented for a threshold probability of 0.5.

| Fold | AUC | PPV | Sensitivity | Specificity | F1 | Accuracy |
| --- | --- | --- | --- | --- | --- | --- |
| *1* | 0.88 | 0.86 | 0.87 | 0.73 | 0.87 | 0.82 |
| *2* | 0.87 | 0.86 | 0.86 | 0.73 | 0.86 | 0.81 |
| *3* | 0.88 | 0.79 | 0.94 | 0.52 | 0.86 | 0.79 |
| *4* | 0.83 | 0.82 | 0.89 | 0.64 | 0.86 | 0.80 |
| *5* | 0.90 | 0.83 | 0.94 | 0.64 | 0.88 | 0.83 |
| *6* | 0.91 | 0.88 | 0.95 | 0.76 | 0.92 | 0.89 |
| *7* | 0.86 | 0.87 | 0.83 | 0.76 | 0.85 | 0,80 |
| *8* | 0.88 | 0.84 | 0.92 | 0.67 | 0.88 | 0.83 |
| *9* | 0.84 | 0.85 | 0.82 | 0.74 | 0.84 | 0.79 |
|  |  |  |  |  |  |  |
| **overall** | 0.87 | 0.84 | 0.89 | 0.69 | 0.87 | 0.82 |

Table S4: Neural Net (Nested) Cross-validation Performances Summary for repeat ablation prediction based on first ECG (within 1 month) after ablation. The PPV, Sensitivity, Specificity, F1, and Accuracy are presented for a threshold probability of 0.4.

| Fold | AUC | PPV | Sensitivity | Specificity | F1 | Accuracy |
| --- | --- | --- | --- | --- | --- | --- |
| *1* | 0.65 | 0.58 | 0.20 | 0.95 | 0.30 | 0.76 |
| *2* | 0.66 | 0.62 | 0.23 | 0.95 | 0.33 | 0.77 |
| *3* | 0.58 | 0.50 | 0.14 | 0.95 | 0.22 | 0.74 |
| *4* | 0.62 | 0.43 | 0.17 | 0.92 | 0.25 | 0.73 |
| *5* | 0.54 | 0.39 | 0.14 | 0.92 | 0.21 | 0.72 |
| *6* | 0.74 | 0.50 | 0.17 | 0.96 | 0.27 | 0.76 |
| *7* | 0.63 | 0.67 | 0.17 | 0.97 | 0.27 | 0.77 |
| *8* | 0.64 | 0.54 | 0.21 | 0.94 | 0.30 | 0.76 |
| *9* | 0.58 | 0.36 | 0.15 | 0.91 | 0.21 | 0.72 |
|  |  |  |  |  |  |  |
| **overall** | 0.62 | 0.51 | 0.18 | 0.94 | 0.26 | 0.75 |

Table S5: Neural Net (Nested) Cross-validation Performances Summary Validation. The PPV, Sensitivity, Specificity, F1 and Accuracy are presented for a repeat ablation threshold probability of 0.275.

| Fold | AUC | PPV | Sensitivity | Specificity | F1 | Accuracy |
| --- | --- | --- | --- | --- | --- | --- |
| *1* | 0.65 | 0.32 | 0.37 | 0.82 | 0.34 | 0.73 |
| *2* | 0.66 | 0.31 | 0.35 | 0.82 | 0.32 | 0.73 |
| *3* | 0.65 | 0.33 | 0.41 | 0.81 | 0.37 | 0.73 |
| *4* | 0.62 | 0.28 | 0.35 | 0.79 | 0.31 | 0.71 |
| *5* | 0.64 | 0.39 | 0.32 | 0.88 | 0.36 | 0.78 |
| *6* | 0.63 | 0.32 | 0.21 | 0.90 | 0.26 | 0.77 |
| *7* | 0.63 | 0.27 | 0.37 | 0.77 | 0.31 | 0.70 |
| *8* | 0.63 | 0.26 | 0.32 | 0.79 | 0.29 | 0.70 |
| *9* | 0.62 | 0.31 | 0.20 | 0.89 | 0.24 | 0.76 |
|  |  |  |  |  |  |  |
| **overall** | 0.63 | 0.31 | 0.32 | 0.83 | 0.31 | 0.73 |

Table S6: Neural Net (Nested) Cross-validation Performances Summary Training. The PPV, Sensitivity, Specificity, F1 and Accuracy are presented for a repeat ablation threshold probability of 0.275.

| Fold | AUC | PPV | Sensitivity | Specificity | F1 | Accuracy |
| --- | --- | --- | --- | --- | --- | --- |
| *1* | 0.70 | 0.42 | 0.46 | 0.85 | 0.44 | 0.78 |
| *2* | 0.70 | 0.42 | 0.46 | 0.85 | 0.44 | 0.78 |
| *3* | 0.66 | 0.39 | 0.42 | 0.85 | 0.41 | 0.77 |
| *4* | 0.69 | 0.39 | 0.50 | 0.82 | 0.44 | 0.76 |
| *5* | 0.66 | 0.41 | 0.35 | 0.88 | 0.38 | 0.78 |
| *6* | 0.69 | 0.50 | 0.36 | 0.92 | 0.42 | 0.81 |
| *7* | 0.71 | 0.36 | 0.49 | 0.80 | 0.42 | 0.74 |
| *8* | 0.68 | 0.36 | 0.49 | 0.80 | 0.42 | 0.75 |
| *9* | 0.63 | 0.42 | 0.29 | 0.91 | 0.34 | 0.79 |
|  |  |  |  |  |  |  |
| **overall** | 0.72 | 0.40 | 0.43 | 0.85 | 0.41 | 0.77 |
